# Expression and diagnostic values of calretinin and CK5/6 in cholangiocarcinoma

**DOI:** 10.1186/2162-3619-3-12

**Published:** 2014-04-23

**Authors:** Lanjing Zhang, Renee Frank, Emma E Furth, Amy F Ziober, Virginia A LiVolsi, Paul J Zhang

**Affiliations:** 1 Department of Pathology and Laboratory Medicine, University of Pennsylvania Pearlman School of Medicine, Philadelphia, PA, USA; 2 Departments of Pathology, University Medical Center of Princeton at Plainsboro/Rutgers Robert Wood Johnson Medical School, Plainsboro, NJ, USA; 3 Department of Chemical Biology, Ernest Mario School of Pharmacy, Department of Pathology and Lab Medicine, Robert Wood Johnson Medical School, and Cancer Institute of New Jersey, Rutgers University, Piscataway, NJ, USA; 4 Department of Pathology, 6 Founders, 3400 Spruce St, Philadelphia, PA 19104, USA

**Keywords:** Cholangiocarcinoma, Calretinin, CK5/6 and immunohistochemistry, Differentiation

## Abstract

**Background:**

Mesothelin, a mesothelial marker, has been found expressed in and as a potential treatment target of cholangioacarcinoma (CC). It is possible that CC may be derived from the cells sharing mesothelial markers. However, the expression of other mesothelial markers in CC is largely unknown.

**Methods:**

Thirty CC cases (10 extrahepatic and 20 intrahepatic) were retrieved from our institutional archive. The immunohistochemical study of Calretinin (DC8), WT1 (6F-H2), Lymphatic Endothelial Marker (D2-40), CK5/6 (D5/16 B4) and CK19 (b170) was done on formalin fixed paraffin embedded sections for 2–3 blocks of each case. We compared the expression levels between CC and normal bile duct (NBD) on the same block.

**Results:**

All of the CC and NBD are positive for CK19 (23/23) and negative for WT1 (0/23) and D2-40 (0/23), except one CC positive for D2-40(1/30, 3.3%) and one NBD positive for WT1 (1/23, 4.3%). Calretinin immunoreactivity was detected in 52.2% (12/23) of CC, but none in NBD (0/23). CK5/6 was also detectable in 73.3% (22/30) of CC and all NBD (30/30). Increased expression of calretinin and reduced expression of CK5/6 were more likely associated with CC than NBD (*P* < 0.001 and *P* = 0.002, respectively). The sequential staining pattern of positive calretinin and negative CK5/6 in calretinin negative cases has a sensitivity of 69.57% and a specificity of 100% for differentiating CC from NBD. CK5/6 expression was also more likely associated with well-differentiated CC (7/7 versus 12/20 in moderately differentiated, and 9/10 in poorly differentiated, *P* = 0.019) and extrahepatic CC (10/10 versus 12/20 in intrahepatic, *P* = 0.029), but there was no association between the calretinin expression and the CC grade or location.

**Conclusion:**

Calretinin and CK5/6 immunohistochemical stains may be useful for diagnosing a CC. Their immunohistochemical results should be interpreted with caution in the cases with differential diagnoses of mesothelioma and CC. A full mesothelioma panel, including WT1 and/or D2-40, is recommended to better define a mesothelial lineage. The biology of calretinin and CK5/6 expression in CC is unclear, but might shed light on identifying therapeutic targets for CC.

## Introduction

Intrahepatic cholangiocarcinoma (CC) is a relatively rare carcinoma of the biliary tree, with rising incidence and mortality [1,2]. Its 1-, 2- and 5-year survivals in US are 24.5%, 11.8% and 3.2%, respectively [1]. The diagnosis and prognostication of CC become critical for managing those patients. Studies have shown that several immunohistochemical (IHC) markers are highly expressed in CC including Annexin A1 (94.1%), CK19 (89%), MOC31 (88.2%), CK7 (83.4%), CD133 (79%), claudin4 (69.2%), high mobility group A1 (HMGA1) (31.5%) and S100P [3-6], while others has no or very low expression in CC such as glypican 3 (GPC3) (7%) and biglycan (7%) [3]. However, the markers’ expression levels are rarely compared with that of normal bile duct (NBD) which could be a morphologic mimic for CC in small lesion or small sampling. In addition to pancreatic carcinoma, a recent study shows that mesothelin, a mesothelial marker, is also found in 33% of resected CC specimens, but not hepatocellular carcinoma (HCC) or normal liver tissue [7]. Moreover, mesothelin may be used as a target for monoclonal antibody therapy in a subset of CC in mice and as a prognostic factor for CC [8,9]. It is possible that mesothelium related proteins and/or genes may also be present in other tumors and involved in their tumorigenesis. Indeed, mesothelin and calretinin are found expressed in thymic carcinoma, thymoma, and non-keratinizing squamous cell carcinoma of lung [10,11]. But little is known regarding the expression levels, if any, of other mesothelial markers such as calretinin, CK5/6, D2-40 and WT1. Hence, we aimed to examine the IHC staining pattern of those markers in CC and NBD, and to explore the potential “mesothelial” phenotype in CC. The findings may help identify more diagnostic markers and therapeutic targets for CC.

## Materials and methods

Histologically and clinically well documented CC cases were identified and retrieved from our institutional archive. The inclusion criteria included: 1. diagnosis of cholangiocarcinoma could be confirmed by review of the slides; 2. A primary tumor must clinically and pathologically arise within the hepatobiliary system, 3. patient had no past and current history of tumors in other system, 4. It was a resection specimen and had at least 3 blocks with carcinoma available for immunohistochemical (IHC) stains; 5. It had both CC and nearby NBD present on the same slide (A control H&E slide was made to confirm this, after being cut for IHC stains).

The IHC protocol and related antibodies have been described before [12]. Briefly, the IHC stains of M2A antigen (clone D2-40, 1:25, Signet Laboratories, Dedham, MA), WT1 (clone 6F-H2, 1:400, DakoCytomation, Carpinteria, CA), calretinin (DC8, 1:50, Zymed Laboratories, South San Francisco, CA), and cytokeratin 5/6 (clone D5/16 B4, 1:25, DakoCytomation) and CK19 (clone b170, 1:100, Leica/Novocastra) were conducted on formalin fixed paraffin embedded tissue sections with standard IHC protocols (BondMax, Leica Microsystem, Buffalo Grove, IL) on 2–3 blocks of each case. Appropriate positive and negative controls were performed and validated.

The protein expression levels were independently assessed by two of the authors (LZ and RF). When a disagreement was present, the two would have a discussion, consult with the senior author (PJZ) and reach an agreement upon re-review of the case. A scoring scale of 0–3 was used, with 0 for negative, 1+ for <25%, 2+ for 26-75%, and 3+ for >75%. The staining intensity was not considered for the purpose of scoring the stains. The literature search for related IHC markers’ positive rates was performed by using the marker’s names and tumors as the search term in Pubmed (NCBI, NIH, USA) in Feb. 2014.

The statistical analyses were performed by using Stata (version 11, StataCorp LP). The 95% confidence intervals (CI) were calculated by using normal distribution. Pearson Chi-square test was used to determine the association between IHC markers and tissue samples, so was Fisher exact test as a confirmation for groups with case number fewer than 6. All of the *P* values were calculated for 2-sided. A *P* value less than 5% was considered statistically significant.

## Results

A total of 30 CC cases (10 extrahepatic and 20 intrahepatic) with nearby NBD met the inclusion criteria and were included in this retrospective study. They were collected between 2005 and 2011. The stained slides from the 2 to 3 blocks of each case all showed similar IHC staining pattern for each marker evaluated. The IHC stains for D2-40 and CK5/6 were performed on all of the 30 cases, and for calretinin, CK19 and WT-1 on 23 of the 30 cases due to the difficulty in obtaining additional blank slides after the dropped tissue sections in the first 2 IHC attempts. As shown in Table 1, the CC and NBD of all cases (23/23, 100% for both) were positive for CK19, a known pancreatobiliary marker. All CC and NBD were negative for WT-1 and D2-40, both known as mesothelial markers, except 1 NBD positive for WT-1 (1/23, 4.3%) and 1 CC positive for D2-40 (1/30, 3.3%). Those unexpected positive WT-1 and D2-40 stains were focal, and scored only 1+ (less than 25%, see Table 2). No significant difference of CK19, WT-1 or D2-40 IHC stain was found between the CC and NBD groups (Tables 1 and 2).

**Table 1:** Immunohistochemical profiles of the cholangiocarcinoma and accompanying normal bile ducts

		**Negative, n (mean%, 95% confidence intervals)**	**Positive, n (mean%, 95% confidence intervals)**	**Total, n (%)**	***P *** value*	***P *** **value#**
Calretinin	CC	11 (47.8, 26.8-69.4)	12 (52.2, 30.6-73.2)	23 (100)	<0.001	NA
	NBD	23 (100, 85.2-100^)	0 (0)	23 (100)		
WT1	CC	23 (100, 85.2-100^)	0 (0)	23 (100)	0.312	1
	NBD	22 (95.6, 78.1-99.9)	1 (4.3, 0.1-21.9)	23 (100)		
D2-40	CC	29 (96.7, 82.8-99.9)	1 (3.3, 0–17.2)	30 (100)	0.313	1
	NBD	30 (100, 88.4-100^)	0 (0)	30 (100)		
CK19	CC	0 (0)	23 (100, 85.2-100^)	23 (100)	0.312	NA
	NBD	0 (0)	23 (100, 85.2-100^)	23 (100)		
CK5/6	CC	8 (26.7, 12.3-45.9)	22 (73.3, 54.1 -87.7)	30 (100)	0.002	NA
	NBD	0 (0)	30 (100, 88.4-100^)	30 (100)		

Note: CC: Cholangiocarcinoma, NBD: accompanying normal bile duct, NA: not applicable, *comparison between CC and NBD by using Pearson Chi-square test, #comparison between CC and NBD by by using Fischer’s exact test, ^one-sided, 97.5% confidence interval.

**Table 2:** Immunohistochemical stain scores of the cholangiocarcinoma and accompanying normal bile ducts

	**Calretinin, n (%)**		**WT1, n (%)**		**D2-40, n (%)**		**CK19, n (%)**		**CK5/6, n (%)**	
	**CC**	**NBD**	**CC**	**NBD**	**CC**	**NBD**	**CC**	**NBD**	**CC**	**NBD**
Negative	11 (47.8)	23 (100)	23 (100)	22 (95.6)	29 (96.7)	30 (100)	0 (0)	0 (0)	8 (26.7)	0 (0)
1+	4 (30.8)	0 (0)	0 (0)	1 (4.3)	1 (3.3)	0 (0)	0 (0)	0 (0)	10 (33.3)	8 (26.7)
2+	4 (30.8)	0 (0)	0 (0)	0 (0)	0 (0)	0 (0)	1 (4.3)	0 (0)	4 (13.3)	7 (23.3)
3+	4 (30.8)	0 (0)	0 (0)	0 (0)	0 (0)	0 (0)	22 (95.6)	23 (100)	8 (26.7)	15 (50)
All Positive	12 (52.2)	0 (0)	0 (0)	1 (4.3)	1 (3.3)	0 (0)	23 (100)	23 (100)	22 (73.3)	30 (100)
total	23 (100)	23 (100)	23 (100)	23 (100)	30 (100)	30 (100)	23 (100)	23 (100)	30 (100)	30 (100)

Note: CC: Cholangiocarcinoma, NBD: accompanying normal bile duct.

**Figure 1:**
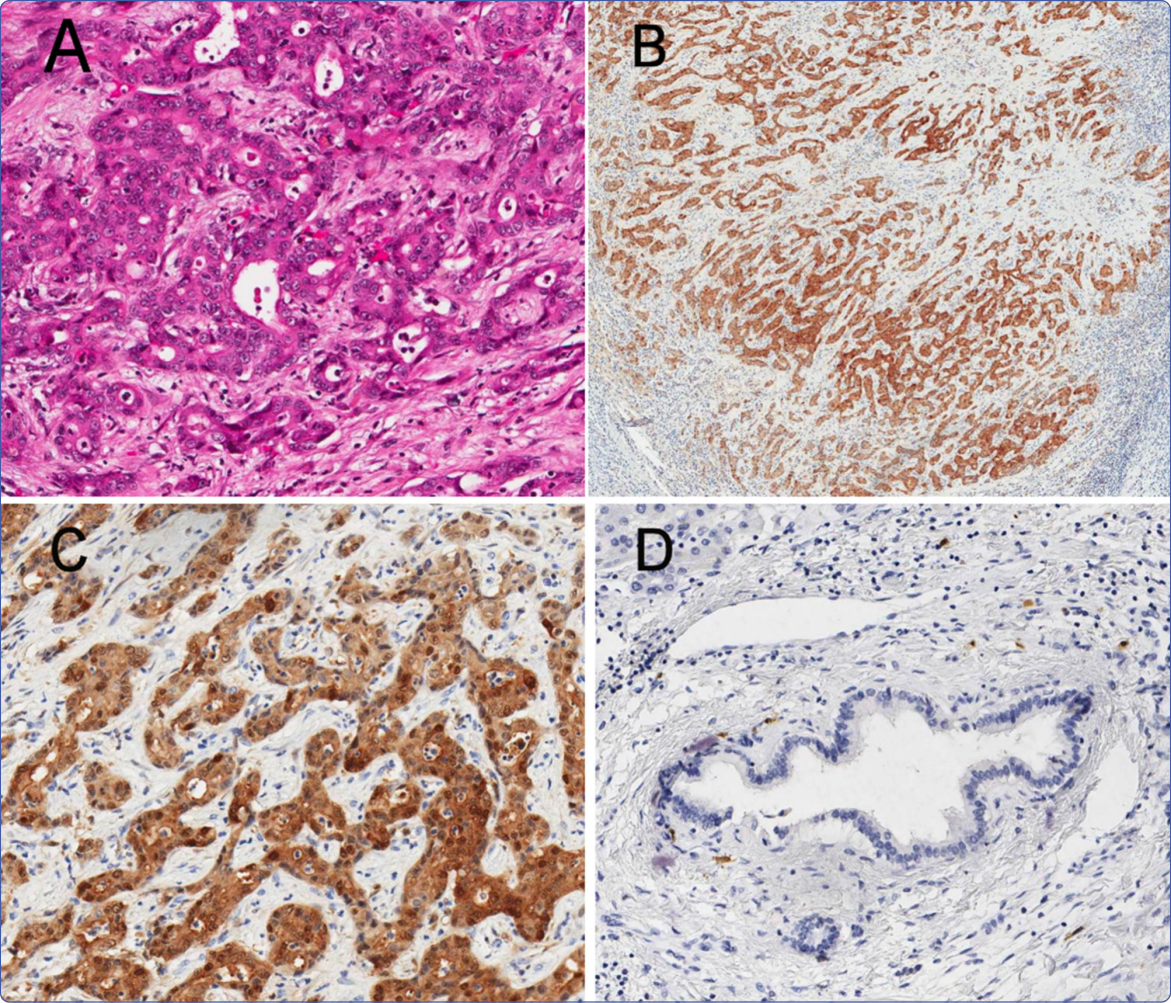
A case showed nuclear and cytoplasmic calretinin reaction in CC (A: H&E, B&C: calretinin ×40& ×200), and a negative cytoplasmic stain in NBD (D: calretinin ×200).

**Figure 2:**
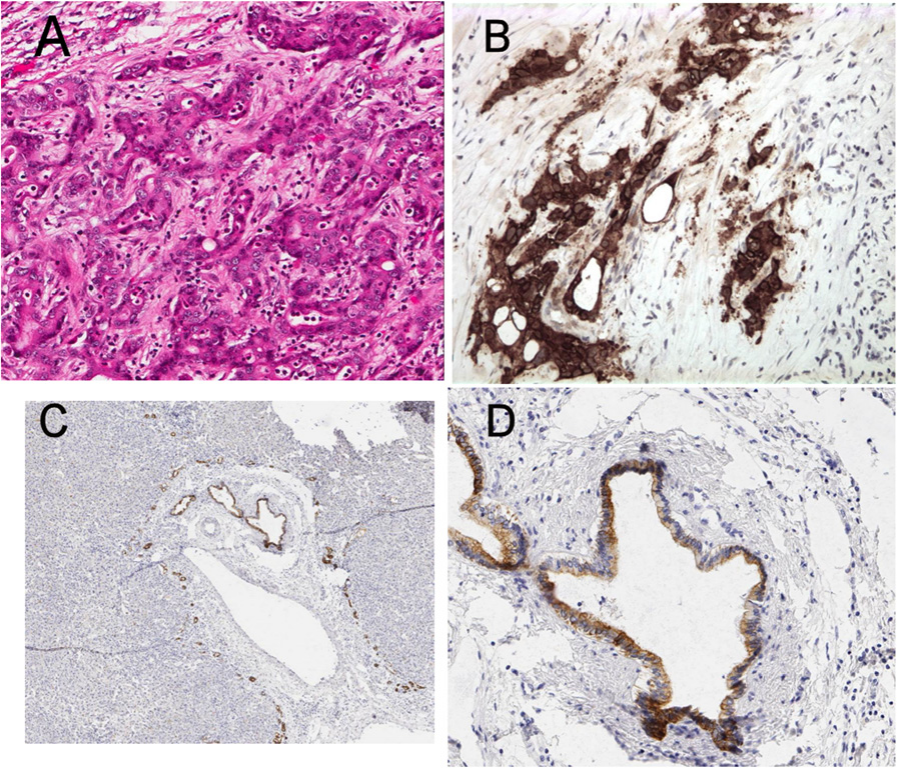
A case showed nuclear and cytoplasmic CK5/6 reaction in CC (A: H&E, B: CK5/6 ×200) and a weaker cytoplasmic stain in a NBD (C&D: CK5/6 ×40& ×200).

Of the 23 CC cases evaluated for calretinin positivity, 12 (52.2%, 95% CI 30.6-73.2%) were stained positive. CC tumor cells of the 12 cases showed dense and diffuse nuclear and cytoplasmic staining pattern of calretinin (Figure 1), while negative in the adjacent NBD in all cases (*P* < 0.001). For CK5/6 evaluation, 22 (73%) of CC and all 30 (100%) of NBD were positive (Table 2, Figure 2). Comparison of the CK5/6 expression in CC and the adjacent NBD revealed CC stained weaker than NBD in 18 (60.0%) cases, similar to NBD in 10 (33.3%), and stronger than NBD in 2 (6.67%) (Tables 2 and 3). Eight out of the 30 CC cases (26.7%, 95% CI 12.3-45.9%) were stained negative for CK5/6 while none of the NBD was negative (*P* = 0.002). The difference of CK5/6 in CC versus adjacent NBD is statistically significant (*P* = 0.022, Table 3). Of note, the two cases with CC stained stronger than NBD had 3+ stains in CC and 2+ in NBD (25–50 staining area difference). In the meantime, 9 (50%) of the 18 cases with CC stained weaker than NBD presented a difference of 2+ or more (50-99%) (Table 3).

**Table 3:** Comparison of CK5/6 stains between cholangiocarinoma and normal bile duct

	**CC**			
NBD	0	1	2	3
0				
1	3	4		
2	**3**	2		2
3	**2**	**4**	4	6

Note: Stain intensity comparison: CC < NBD: 18, CC = NBD: 10, and CC > NBD: 2 (*P* = 0.022); CC: Cholangiocarcinoma, NBD: accompanying normal bile duct. The number in bold shows the case number with stain difference of more than 1+ (>25% difference).

We then sought the best IHC diagnostic criteria for differentiating CC versus NBD by comparing various cutoff values of the 2 markers (Table 4). The best sensitivity of 69.59% was achieved by using the sequential stains of calretin and CK5/6, with positive calretinin and negative CK5/6 in the calretinin negative cases as the positive result for CC (sequential calretinin and CK5/6 criteria), while maintaining 100% specificity. This sensitivity was statistically higher than that of calcretinin stain more than 2+, or negative CK5/6 stain. However, we did not find the sensitivity difference between the sequential calretinin and CK5/6 criteria and the others, including positive calretinin stain alone (52.17%), CK5/6 stained negative or 1+ (60.0%) and less CK5/6 stain in CC than NBD (56.67%) (Table 4). Our Fisher exact test also revealed that CK5/6 expression was more likely associated with well-differentiated CC and extrahepatic CC, but no association between the calretinin expression and the CC grade or location (Table 5).

**Table 4:** Comparison of various calretinin and CK5/6 diagnostic criteria for cholangiocarcinoma

**No.**	**Criteria**	**CC**	**Total CC**	**Sensitivity**	***P *** value*	**NBD**	**Total NBD**	**Specificity**	***P *** value*
1	Cal 1+	12	23	52.17%	0.227	23	23	100.00%	NA
2	Cal 2+	8	23	34.78%	0.018	23	23	100.00%	NA
3	Cal 3+	4	23	17.39%	<0.001#	23	23	100.00%	NA
4	CK5/6 -	8	30	26.67%	0.002	30	30	100.00%	NA
5	CK5/6 - or 1+	18	30	60.00%	0.472	22	30	73.33%	0.007
6	Less CK5/6 staining in CC than in NBD	17	30	56.67%	0.337			NA	
7	Cal+, and CK5/6 - in Cal - cases	16	23	69.57%		23	23	100.00%	

Note: Cal: calretinin, CC: Cholangiocarcinoma, NBD: accompanying normal bile duct, NA: not applicable, *Comparison between criteria 7 and other criteria by using Pearson Chi-square test, #Comparison between criteria 7 and 5 by using Fischer’s exact test.

**Table 5:** Association of the cholangiocarcinoma grades and locations with calrectinin and CK5/6 expression

		**Calretintin**				**Calretinin**						**CK 5/6**			**CK5/6**			
	**Neg**	**1+**	**2+**	**3+**	***P***	**Neg**	**Pos**	**Sum**	***P***	**Neg**	**1+**	**2+**	**3+**	***P***	**Neg**	**Pos**	**Sum**	***P***
WD	3	1	2	1	#	3	4	7	#	0	2	3	2	0.032	0	7	7	0.019
%	42.86	14.29	28.57	14.29		43	57.1	100		0	28.57	42.86	28.57		0	100	100	
MD	6	2	2	0		6	4	10		7	4	0	2		7	6	13	
%	60	20	20	0		60	40	100		53.85	30.77	0	15.38		53.85	46.15	100	
PD	2	1	0	3		2	4	6		1	4	1	4		1	9	10	
%	33.33	16.67	0	50		33	66.7	100		10	40	10	40		10	90	100	
EH	3	0	3	1	#	3	4	7	#	0	4	0	6	0.004	0	10	10	0.029
%	42.86	0	42.86	14.29		43	57.1	100		0	40	0	60		0	100	100	
IH	8	4	1	3		8	8	16		8	6	4	2		8	12	20	
%	50	25	6.25	18.75		50	50	100		40	30	20	10		40	60	100	
Sum	11	4	4	4		11	12	23		8	10	4	8		8	22	30	
%	47.83	17.39	17.39	17.39		48	52.2	100		26.67	33.33	13.33	26.67		26.67	73.33	100	

Notes: WD = well-differentiated, MD = moderately-differentiated, PD = Poorly-differentiated, EH = extrahepatic cholangiocarcinoma, IH = intrahepatic cholangiocarcinoma, *P*: *P*-value, #: *P* > 0.005.

## Discussion

Cholangiocarcinoma is an uncommon carcinoma in the developed countries, but had a rising mortality in both UK and USA [1,2]. Its carcinogenesis and diagnostic markers are not well defined. Studies have revealed some IHC markers such as CK7, CK19, MOC31, claudin4, HMGA, CD133 and Annexin A1, but with variable specificities (Table 6) [3-5,13]. In particular, little is known about the IHC marks’ expression in NBD. To our best knowledge, this study is the first to explore the use of a set of known mesothelial markers for differentiating CC from accompanying NBD.

**Table 6:** Positive rates of immunohistochemical markers for mesothelioma, cholangiocarcinoma, lung adenocarcinoma and hepatocellular carcinoma

	**Mesothelioma (all subtypes included)**	**Cholangiocarcinoma**	**Lung adenocarcinoma**	**Hepatocellular carcinoma**
WT-1	43-93% [11,12,14-17]	0%#	0-7% [12,14,16]	NA
D2-40	86-100% [12,18,19]	3%#	0-33% [12,18,19]	NA
Calretinin	92.4-100% [11,12,16,17,20,21]	52%#	8-23% [12,16,22]	NA
CK5/6	64-100% [11,12,15-17,20]	0-73.3%#, [23]	0-39% [12,14-16,23]	NA
Mesothelin	47-100% [14,16,17]	33% [7]	38-100% [14,16,22]	NA
CK19	NA	89-100#, [3,24]	NA	2-10.1% [3,24]
Annexin A1	NA	94.1% [5]	NA	0% [5]
Glypican-3	NA	6-7% [3,24]	3.6-9.6% [25,26]	69-87.1% [3,24-26]
Arginase	NA	0% [24]	0% [26]	94-95% [24,26]
TTF-1	0% [16]	0-10% [23,27]	(nuclear) 20-74% [16,23]	(cytoplasmic) 50-93% [27-29]
HepPar-1	NA	0-7% [23,24]	8.1% [26]	74-100% [23,24,26]
MOC31	8-35% [14,16]	88.2% [3]	92-100% [14,16,17,30]	34.0% [3]
EMA	79-93% [16,21]	100% [31,32]	100% [16]	12.5-23% [31,32]
BG 8	7% [16]	NA	95-96% [16,17]	NA
BerEP4	16-18% [16,21]	100% [23]	74-100% [16,17]	33% [23]

Note: #indicates this study; Sensitivity reported in some studies [17] is included; NA: No IHC data on the respective marker are identified by searching the Pubmed database.

Calretinin is a 29–30 kilodalton calcium binding protein primarily expressed in the nerves [33]. Since it was found expressed in mesothelioma in 1990s [34], many types of tumor and tissue are found immunocreactive to calretinin including 22.5% of examined colonic carcinomas [35], 81.5% of ameloblastomas [36], 36% of thymic carcinomas [10], 100% of cardiac myxomas [37], 56-100% sex cord-stromal and 90-100% fibrous neoplasms of the ovaries [38-40], 95% olfactory neuroblastoma [41], 95% of adrenal cortical tumors [42], 71% of synovial sarcomas [43], 15% breast carcinomas [44], skin [45] and others [46]. Our study indicates that CC may be calretinin positive regardless of CC grade and location, and should be considered in the differential diagnoses for calretinin positive tumor. As many as 52% of our CC cases showed strong nuclear and cytoplasmic calretinin expression, while none in the NBD, suggesting a potential role of calretinin in differentiating CC from NBD and in CC carcinogenesis (Table 4). In contrast, CK19 positivity does not discriminate CC and non-cancerous NBD, and should be used only for confirmation of a bile duct (biliary) lineage.

We also examined the expression of other mesothelial markers in CC and NBD including WT1, D2-40 and CK5/6 [12,30,47-49]. Not surprisingly, our data showed that WT1 and D2-40 remained highly specific for mesothelial lineage, and should be included in the panel to differentiate mesothelioma from CC. Of note, WT1 has also been reported positive in more than 90% in adenomatoid tumors [50], 76% ovarian sex cord-stromal tumor [39], 77% serous papillary carcinoma of the ovary [40], and 29% of endometrioid carcinoma [40]. Caution therefore should be used when interpreting a WT1 positive stain, particularly in female patients. Addition of D2-40 may also improve the sensitivity and specificity for confirming mesothelial differentiation [12].

CK5/6 is a high molecular-weight cytokeratin highly expressed in stratified epithelium and mesothelium, first found useful for distinguishing mesothelioma from adenocarcinoma in late 1990s [51,52]. Its positive rate in mesothelioma is comparable to that of calretinin [20]. A recent systemic review also confirms that CK5/6 is one of the two most sensitive IHC markers (sensitivity of 83%), and one of the two most specific IHC markers (specificity of 85%) for epitheloid mesothelioma [30]. However, many tumors other than mesothelioma are also positive for CK5/6, including but not limited to 88% of adenosquamous carcinoma of the pancreas [53], 55% of metastatic squamous carcinoma of various origin [54], 75% of lung squamous cell carcinoma in fine needle aspirate specimens [55], 100% of squamous cell carcinoma in pleural fluids [14], 98% of squamous cell carcinoma and 18% of adenocarcinoma in the lung [56], 62% of urothelial carcinoma [57], and 50% of endometrial adenocarcinoma [57]. Our study showed that all NBD and 73.3% of CC were positive for CK5/6. Further analysis found that CK5/6 expression was significant lower in the CC than the accompanying NBD (Table 3). Those findings demonstrate the differential expressions of CK5/6 in CC and NBD, and suggest a potential use of CK5/6 in differentiating CC from NBD. The association between CK5/6 expression and extrahepatic and well-differentiated CC indicates a preference of using CK5/6 in those CCs.

Of the markers we tested, the best sensitivity to identify CC is reached by using sequential criteria of positive calretinin and negative CK5/6 in the calretinin negative cases, with a 100% specificity. Using positive calretinin stain criterion is straight forward, and will result in only a 17.47% loss of sensitivity, or missing 4 out of 23 cases as shown in our study. This may be the second best strategy of using calretinin and/or CK5/6 to differentiate CC from NBD. The criterion of less CK5/6 stain in tumor than NBD requires presence of both tumor and NBD in the same specimens, which would be particularly challenging in core biopsies and cytology specimens. We therefore doubt its clinical usefulness due to specimen type limitations. Negative or 1+ CK5/6 stain gave us the second highest sensitivity, but also led to a significantly lower specificity (100% vs 73.3%, *P* = 0.007). Despite its single IHC stain requirement, we felt the lower specificity would be a serious concern for its clinical use. Together, we recommend sequential staining pattern of positive calretinin and negative CK5/6 in the calretinin negative cases for differentiating CC from NBD. Future work may focus on larger-scale studies and differentiating CC from HCC and mesothelioma by using these criteria.

We summarized the expression profiles of the common IHC markers for CC, mesothelioma and HCC in Table 6. Interestingly, the positive rates of those markers vary among studies. For example, the positive rate of MOC31 in mesothelioma ranged from 8% to 35%. Consistent with our summary, an excellent review and another guidelines have confirmed such a variation among reports, and made the practical yet useful recommendations on how to best utilize those markers [15,48,58]. We suggest to include at least WT1 and D2-40 in the panel to confirm a mesothelial lineage, with optional addition of calretinin, CK5/6 and/or mesothelin. Should WT1 and/or D2-40 stain be not interpretable due to technical issues or limitations (such as a fallen section), one must use other mesothelial markers to rule out the tumors with calretinin and CK5/6 reactivity from mesothelioma, such as CC as shown in this study. The reported different positive rates of the IHC markers may be in part attributed to the various IHC staining methods. For example, we noticed that CK5/6 expression was present in 2% of lung adenocarcinoma by using 1:25 dilution of D5/16B4 antibody (Boehringer-Mannheim) and Envision + biotin free detection system in Dako AutoStainer [16], but 39% by using the same 1:25 dilution of different D5/16B4 antibody (Dako) and Envision + HRP detection system in the same IHC stainer [12]. Similarly, using different dilutions of calretinin antibody (rabbit, Zymed, South San Francisco, CA) seemed to result in different calretinin positive rates in lung adenocarcinoma (8% versus 23%) [11,12,16]. Another study also showed that the TTF-1 positive rates in hepatocellular carcinomas vary from 0% to 70% depending on the antibody manufacturer [28]. An in-house validation of new antibodies on various tumors seems a reasonable safe-guard approach and is recommended.

On the cancer biology level, calretinin has higher positive rates in CC than mesothelin (52.17% versus 33%), and may also be a more sensitive and/or specific therapeutic target for CC. However, little is known regarding the roles of calrectin and CK5/6 in the carcinogenesis of CC and the biology of biliary epithelium. Given the recent identification of both mesothelial progenitor cells and liver stem cells [59-62], we hypothesize that the expression of calretinin and CK5/6 in CC is an aberrant differentiation of liver/bile duct stem cells, or simply reflecting the partial mesothelial phenotype of the NBD. However, much research is needed to examine our hypothesis.

This study has several limitations. First, this retrospective study may have selection bias and moderate statistical power. Second, the calretinin and CK5/6 staining profiles were not compared between CC, mesothelioma and HCC. A direct comparison of calretinin and CK5/6 expression in those tumors would be more evidential. Third, the calretinin and CK5/6 IHC patterns in ductal proliferation including reactive changes or benign ductal neoplasms were not assessed, but may be of particular value in differentiating carcinoma and non-cancerous lesions. Last, the prognostic value of calretinin and CK5/6 expression in CC is not explored in this study, but may be interesting to investigate because another mesothelial marker, mesothelin, has been considered for therapeutic targets for CC. Survival studies are beyond the scope of this study, however. Future work is needed to address the unanswered questions and our study’s limitations.

## Conclusions

In conclusion, we for first time showed calretinin and CK5/6 expression in CC. The sequential criterion of positive calretinin stain and negative CK5/6 stain in calretinin negative cases has a sensitivity of 69.57% and a specificity of 100% for differentiating CC from NBD. Our data also suggest to include at least one or two markers more specific for mesothelial differentiation, such as D2-40 and WT1, in the panel to define a mesothelial lineage.

## Abbreviations

CC: Cholangiocarcinoma; IHC: Immunohistochemical; GPC3: Glypican 3; NBD: Normal bile duct; CI: Confidence intervals.

## Competing interest

The authors declared that they have no competing interests.

## Authors’ contributions

LZ and PJZ, designed the study, conducted analyses, interpreted results and drafted the manuscript. LZ, AFZ and RF conducted the study and analyses. EFF and VAL reviewed the study design, interpreted results and were involved in manuscript development. All authors read and approved the final manuscript.
